# Majorbio Cloud 2026 provides comprehensive analysis workflows for microbiome

**DOI:** 10.1002/imt2.70109

**Published:** 2026-02-05

**Authors:** Jianhua Zhao, Linmeng Liu, Jichen Han, Junbiao Zhang, Xiaodan Li, Yan Wang, Qianhui Han, Xue Gong, Linna Zhang, Hao Gao, Bing Yang, Yachen Dong, Huasheng Huang, Xianglin Zhang, Kegang Yu

**Affiliations:** ^1^ Shanghai Majorbio Bio‐pharm Technology Co., Ltd Shanghai China

## Abstract

The integrated microbiome data analysis platform on Majorbio Cloud (https://cloud.majorbio.com/) encompasses 26 analytical workflows, with a core architecture of two modules: single‐omics workflows and cross‐omics integration and correlation workflows. The platform supports multi‐scale microbiome research (strain to community levels) and cross‐omics analyses spanning DNA, RNA, protein, and metabolite layers. The platform features four key functions: (1) Application guide, streamlines analytical workflows for user convenience; (2) Default analysis parameters and one‐click analysis, enables one‐step data processing; (3) One‐click plot enhancement, optimizes figures to meet academic publication standards; (4) Plot patchwork feature, stores optimized images in my gallery, facilitates the creation of publication‐ready image composites, supports composite downloads in PDF/PNG/SVG formats, and allows the preservation of patchwork templates for subsequent applications. By late 2025, the platform has facilitated over 5,050 scientific publications, accelerating microbiome research advances.

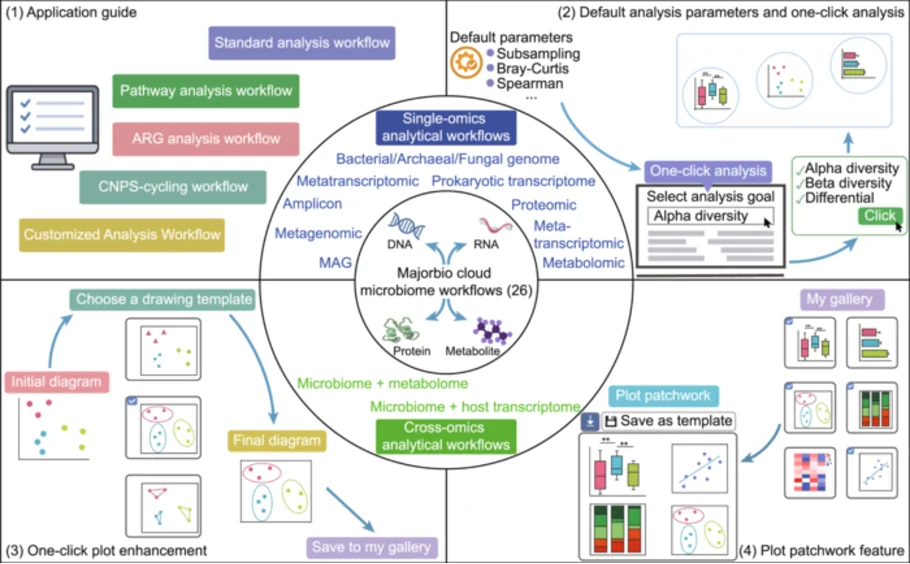

To the editor,

Microorganisms are critical to all life on Earth, playing essential roles in key biological processes and diverse interactions with other organisms that shape ecosystems, drive biogeochemical cycles, and influence both human and environmental health [1]. The rapid advancement of high‐throughput sequencing technologies for environmental samples has revolutionized our understanding of microbial diversity and functions. Vast genomic datasets spanning Earth's biomes now provide a blueprint of microbial life, enabling a more holistic perspective on the structure and function of microbiomes across various ecosystems. Over the last decade, a growing number of computational pipelines have been developed to meet the analytical challenges of high‐throughput sequencing, such as QIIME 2 [2], EasyAmplicon [3], MG‐RAST [4], gcMeta [5], IPGA [6], MicrobiomeAnalyst [7], SAMSA2 [8], metaTP [9], and ViOTUcluster [10].

While existing analytical pipelines have significantly advanced microbiome research in fields such as human health, agriculture, and environmental monitoring, they remain limited in scope and generally lack an integrated cross‐omics perspective (Table S1). Furthermore, most pipelines require users to possess specialized bioinformatics skills, such as coding proficiency for data analysis or the preparation of complex input files, thereby restricting accessibility for non‐specialists. Additionally, the visualization outputs often require manual refinement using professional software (e.g., Adobe Illustrator) prior to publication. As microbiome research advances toward more sophisticated cross‐omics strategies—integrating heterogeneous datasets like microbiome‐metabolome and microbiome‐transcriptome analyses—existing tools often fail to meet the demands of such integrated analyses. To address the growing need for diversified microbiome data analysis, we have developed an integrated platform on the Majorbio Cloud [11, 12]. This platform facilitates multi‐scale research (from strains to communities) and cross‐omics investigations across DNA, RNA, protein, and metabolite layers, incorporating both relative and absolute quantification methods.

All user‐uploaded raw sequencing data and intermediate analysis files are stored securely in our cloud infrastructure, with strict access controls and encryption protocols in place. Users retain full ownership and ultimate management authority over their data. Through the platform's interface, they can manage datasets, control sharing, and assign granular permissions to collaborators.

## INTEGRATED CLOUD PLATFORM FOR MICROBIOME DATA ANALYSIS: AN OVERVIEW

The integrated microbiome data analysis platform comprises a comprehensive suite of 26 analytical workflows. Its core architecture is organized into two primary modules: single‐omics analytical workflows and cross‐omics integration and correlation workflows (Figure 1). This design is engineered to deliver both depth and rigor for in‐depth analysis of individual omics layers, while acting as a bridge to enable high‐dimensional data integration and facilitate biological discovery.

**Figure 1:**
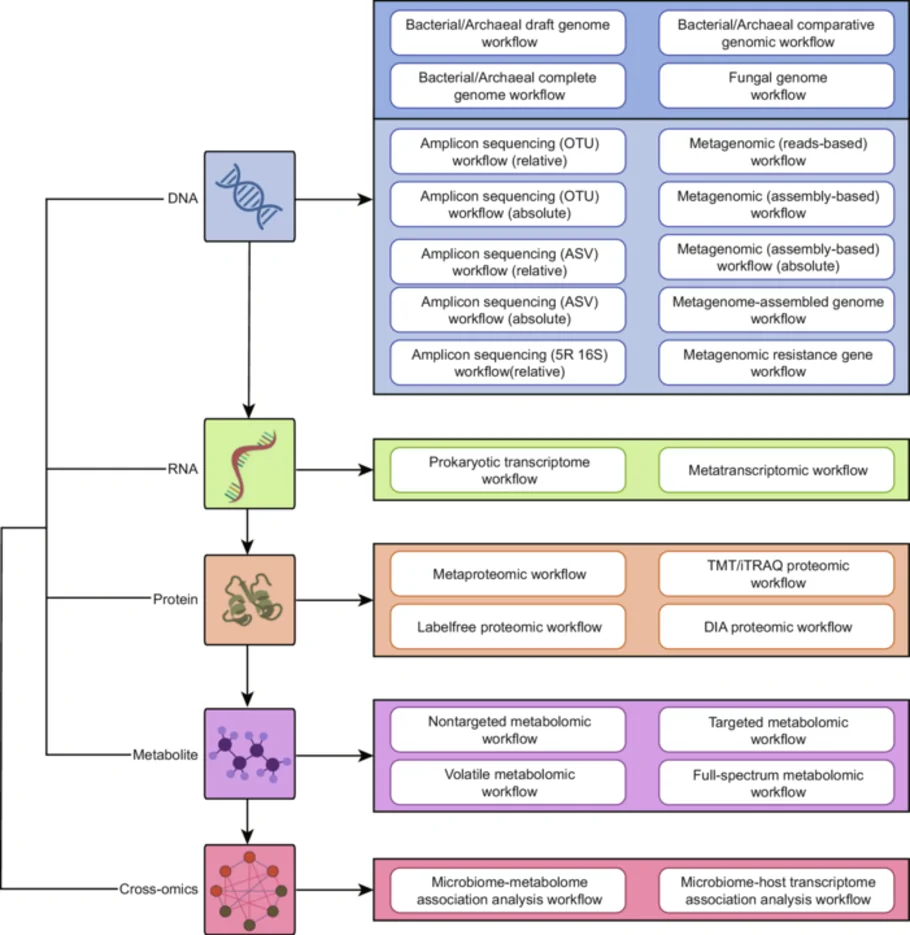
Overview of the integrated cloud platform for microbiome data analysis. The platform encompasses 26 analytical workflows, with its core architecture structured into two main modules: single‐omics analytical workflows and cross‐omics integration and correlation workflows. It supports multi‐scale research (from strains to communities) and comprehensive cross‐omics investigations spanning DNA, RNA, protein, and metabolite layers. Both relative and absolute quantification methods are integrated to facilitate these analyses.

This integrated platform features eight core workflows spanning key domains of microbiome research, enabling precise and in‐depth analysis of each data type: (1) Bacterial (Archaeal)/Fungal genome; (2) Prokaryotic transcriptomics; (3) Amplicon sequencing; (4) Metagenomics; (5) Metagenome‐assembled genome; (6) Metatranscriptomics; (7) Proteomics; and (8) Metabolomics. To overcome the limitations of single‐omics strategies, this platform has incorporated advanced cross‐omics integration workflows, which are specifically designed to elucidate intrinsic correlations across distinct molecular layers and include two core association analysis workflows: (1) Microbiome–metabolome association analysis and (2) Microbiome–host transcriptome association analysis.

A more detailed introduction to the aforementioned workflows is provided in Supporting Information. The software and packages utilized in these workflows are listed in Table S2. A comparative summary of the three binning tools—MetaBAT2, CONCOCT, and MaxBin2—is available in Table S3.

In summary, this integrated platform offers a unified analytical framework for microbiome research, linking descriptive community ecology to the exploration of underpinning microbial mechanisms and providing a solid basis for advancing functional microbiome science.

## CLOUD WORKFLOWS FOR MICROBIOME DATA ANALYSIS

### Example 1: Amplicon sequencing analysis workflow

Amplicon sequencing is a highly targeted approach enabling detailed characterization of specific genomic regions, such as 16S/18S rRNA genes or the Internal Transcribed Spacer (ITS) region. Unlike whole‐genome sequencing, this technique employs PCR‐based amplification of target gene regions prior to sequencing. The analytical workflow is primarily dictated by the data processing paradigm—either clustering reads into Operational Taxonomic Units (OTUs) or resolving exact Amplicon Sequence Variants (ASVs) (Figure 2). Furthermore, the workflow may also vary depending on the sequencing technology utilized (second‐ or third‐generation sequencing platforms) and the quantification strategy adopted (relative or absolute quantification) (Figure 1).

**Figure 2:**
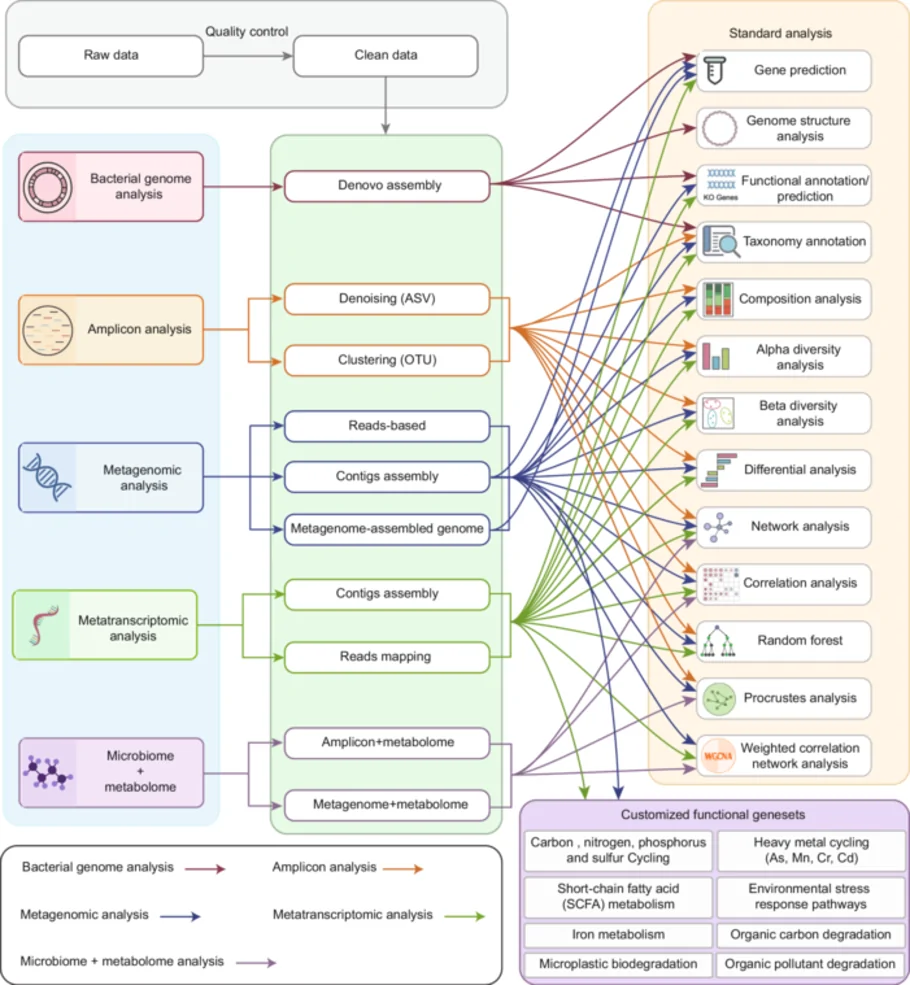
Cloud workflows for microbiome data analysis. This figure features five core analytical workflows: (1) Bacterial genome analysis workflow; (2) Amplicon sequencing analysis workflow; (3) Metagenomic analysis workflow; (4) Metatranscriptomic analysis workflow; (5) Microbiome‐centered cross‐omics analysis workflow. Each workflow incorporates both basic and advanced analytical modules tailored to its specific research objectives.

For data processing, the OTU‐based workflow employs UPARSE [13] for OTU clustering, whereas the ASV‐based workflow utilizes denoising tools such as DADA2 [14], Deblur [15], and UNOISE2 [16]. Taxonomic classification is supported by over 20 accessible taxonomic annotation databases, including SILVA, RDP, Greengenes, NT, UNITE, Protist Ribosomal Reference Database 2 (PR2), MaarjAM, and FunGene. Functional potential prediction is supported via tools such as PICRUSt2, Tax4Fun, BugBase, FAPROTAX, and FUNGuild. The workflow provides 25 alpha diversity indices, covering richness indices (Sobs, Chao1, and Ace), diversity indices (Shannon and Simpson), coverage index (Coverage), evenness index (Pielou's evenness), and phylogenetic diversity index (PD). Beta diversity is explored through hierarchical clustering, Principal Component Analysis (PCA), Principal Coordinate Analysis (PCoA), or Non‐Metric Multidimensional Scaling (NMDS), and the significance of separation is tested by Permutational Multivariate Analysis of Variance (PERMANOVA) or Analysis of Similarities (ANOSIM). Given that microbial communities are often shaped by external environmental factors, the workflow incorporates environmental correlation analysis such as Redundancy Analysis (RDA), distance‐based RDA (db‐RDA), Variance Partitioning Analysis (VPA), and Mantel test.

Furthermore, advanced modules are available for specialized research needs. For microbial community assembly, methods include the Neutral Community Model (NCM), Normalized Stochasticity Ratio (NST), beta Nearest Taxon Index (betaNTI), and infer Community Assembly Mechanisms via Phylogenetic‐bin‐based Null Model Analysis (iCAMP). In medical microbiology research, the workflow integrates predictive modeling approaches such as Random Forest, Support Vector Machine (SVM), k‐Nearest Neighbor (k‐NN), and Least Absolute Shrinkage and Selection Operator (LASSO).

Since its launch in late 2016, the amplicon sequencing analytical workflow has facilitated the publication of approximately 3240 scientific papers (based on a Google Scholar search conducted on December 3, 2025, using keywords: “cloud.majorbio.com operational taxonomic unit OR amplicon sequence variants” or “www.i-sanger.com operational taxonomic unit OR amplicon sequence variants”). This widespread adoption is attributed to the workflow's three core strengths: user‐centric design, scientific rigor, and publication‐ready visualization output.

### Example 2: Metagenomic analysis workflow

Since the concept was first introduced in 1998, metagenomic technologies driven by high‐throughput sequencing have revolutionized our understanding of microbial communities, providing unprecedented insights into the genetic and functional diversity of microorganisms across Earth's ecosystems [17]. The application of long‐read sequencing platforms, such as Oxford Nanopore Technologies (ONT) and Pacific Biosciences (PacBio), enables single‐molecule‐level analysis of microbial communities and their genomic features, thereby delivering near‐complete genomic context. Continuous advancements in bioinformatics data analysis tools are further propelling metagenomic research toward higher resolution and more precise mechanistic interpretations.

Data analysis serves as a cornerstone of metagenomic research. To address diverse analytical demands, this workflow offers three core approaches (Figure 2): read‐based analysis (e.g., Kraken2, MetaPhlAn4, HUMAnN3), assembly‐based analysis (employing tools such as MEGAHIT, IDBA‐UD, SOAPdenovo2), and metagenome‐assembled genome analysis. The following section elaborates on the assembly‐based analysis approach as a representative example.

The assembly‐based metagenomic workflow comprises six sequential core steps: data preprocessing, metagenomic assembly, gene prediction, construction of non‐redundant gene sets, taxonomic and functional annotation, and data visualization with downstream analysis. To support comprehensive investigations, the workflow integrates 20 curated annotation databases, including core databases (e.g., NR, eggNOG, KEGG, CAZy, CARD, VFDB) and specialized databases (e.g., GO, PHI‐base, TCDB, QSDB, Pfam). Furthermore, to cater to diverse research needs, 11 specialized functional gene sets have been curated based on the KEGG database, encompassing key biogeochemical cycles and metabolic pathways: carbon (C) cycling, nitrogen (N) cycling, phosphorus (P) cycling, sulfur (S) cycling, heavy metal cycling (e.g., arsenic, manganese, cadmium, chromium), iron metabolism, environmental stress response pathways, short‐chain fatty acid metabolism (e.g., acetic acid, propionic acid, butyric acid), organic carbon degradation, microplastic biodegradation, and organic pollutant degradation.

Since its initial release in early 2018, this workflow has facilitated the publication of 1687 articles across diverse research fields (based on a Google Scholar search conducted on December 3, 2025, using the search terms: “cloud.majorbio.com metagenome” or “www.i-sanger.com metagenome”). Moving forward, the workflow will be continuously updated and enhanced, integrating more advanced analytical tools and methodologies to improve its usability and analytical depth. It is expected to facilitate efficient and streamlined metagenomic data processing and analysis for an expanding user base.

### Example 3: Microbiome‐centered cross‐omics analysis workflow

Integrated microbiome and metabolome studies represent a paradigm shift from static description to dynamic functional interpretation. This approach goes beyond characterizing microbial composition in isolation, directly revealing how microbial communities interact with their host via metabolites that serve as a “molecular language,” and ultimately elucidating their impacts on host health and disease. Furthermore, integrating microbiome with host transcriptome data enables a systematic dissection of host–microbe interactions, facilitating simultaneous characterization of microbial community structure/function and host gene expression. Such an integrated strategy provides multidimensional evidence for identifying key drivers and biomarkers in studies of disease mechanisms, agricultural ecology, and environmental adaptation.

Statistical and machine learning methods are widely used to analyze paired datasets, such as microbiome–metabolome or microbiome–eukaryotic transcriptome data, to identify microbe–metabolite or microbe–host transcriptome associations. Notably, human gut microbiome–metabolome studies have garnered increasing attention in recent years, driven by accumulating evidence of the interplay among gut microbes, metabolites, and host health [18]. In such gut‐focused investigations, these methods are particularly valuable for identifying specific microbe‐associated metabolites that are potentially modifiable through microbiome‐based interventions, thereby offering a pathway to promote gut metabolic health. Therefore, this section focuses specifically on the integrated microbiome–metabolome analysis workflow as a representative example.

The integrated microbiome‐metabolome analysis workflow enables direct correlation analysis between microbiome and metabolome datasets. Microbiome data typically derive from amplicon sequencing or metagenomic analysis, whereas metabolome data are generated via untargeted or targeted metabolomic profiling (Figure 2). This workflow incorporates 19 analytical approaches, including Procrustes analysis, Two‐way Orthogonal Partial Least Squares (O2PLS), Mantel test, Canonical Correspondence Analysis (CCA), Random Forest, Least Absolute Shrinkage and Selection Operator (LASSO), Logistic Regression, Microbial–Metabolic Interactions Model for Omics data Analysis 2 (MIMOSA2), microbe–metabolite vectors (mmvec), and Weighted Gene Co‐expression Network Analysis (WGCNA).

## CORE FEATURES OF THE INTEGRATED MICROBIOME DATA ANALYSIS CLOUD PLATFORM

### Application guide

The “Application guide” feature (Figure S1) serves as an intelligent, step‐by‐step module tailored for the integrated microbiome data analysis cloud platform. Its primary objective is to minimize the learning curve and maximize data analysis efficiency for users.

The core value of this feature resides in transforming complex bioinformatics workflows into clear, task‐specific guides, thereby significantly accelerating project setup time. This enables users to focus exclusively on scientific inquiry rather than troubleshooting technical details, ultimately delivering a streamlined “one‐click” analytical experience. The module facilitates the rapid configuration of optimized analytical workflows customized to specific research objectives (e.g., biomarker screening, mechanistic pathway exploration). For instance, the metagenomic analysis workflow integrates specialized guides for carbon‐nitrogen‐phosphorus‐sulfur cycling, antibiotic resistance gene profiling, virulence factor analysis, environmental pollutant bioremediation, and key metabolite profiling, allowing users to execute configurations via one‐click automation.

### Default analysis parameters and one‐click analysis

This feature (Figure S1) streamlines data analysis by integrating pre‐optimized default parameters, covering key analytical steps such as sequence subsampling, distance matrix algorithms, correlation methods, and clustering. Preconfigured based on authoritative literature and expert consensus, these parameters thereby ensure scientific validity and reproducibility. Users can initiate analyses without manually adjusting complex parameters; instead, they simply select the appropriate analysis menu based on their research objectives (e.g., alpha diversity analysis, beta diversity analysis, differential analysis), and the one‐click function automates the entire process. This approach offers three core advantages: lowering technical barriers, significantly enhancing analytical efficiency, and ensuring result robustness. Consequently, researchers can focus on addressing core scientific questions rather than navigating technical complexities. Across research, clinical, and industrial settings, this feature accelerates project timelines and facilitates the efficient generation of high‐quality analytical reports.

### One‐click plot enhancement

This feature (Figure S1) is designed to optimize the visualization of microbiome analysis results. It provides intelligent, ready‐to‐deploy, publication‐quality templates for mainstream visualization types, including boxplots, stacked bar charts, heatmaps, PCA plots, PCoA plots, NMDS plots, RDA/CCA plots, db‐RDA plots, linear regression plots, and VPA plots. Each template incorporates pre‐configured color schemes, axis scaling, and label formatting, aligned with the aesthetic standards of top‐tier journals while being tailored to data‐specific characteristics (e.g., sample size, variable distribution). Users simply select the appropriate template to automatically generate visuals with harmonized colors, a clear layout, and emphasized key elements, eliminating the need for manual adjustment of graphic layers or aesthetic parameters. This reduces the barrier to generating high‐quality visualizations, boosts efficiency, and ensures figures are both academically rigorous and visually compelling. Consequently, these research findings effectively engage the audience in manuscripts, reports, or presentations, facilitating the clear dissemination of core findings.

### Plot patchwork feature

This feature (Figure S1) streamlines the assembly and customized layout of multi‐panel figures. Users first save figures generated on analysis pages to the “my gallery” module. The gallery supports comprehensive image management, allowing users to search, organize, and delete images. After selecting a minimum of two figures from the gallery, users enter the composite canvas interface to adjust canvas dimensions, row/column counts, and inter‐figure spacing via layout configuration tools. Images on the canvas can be freely dragged, resized (with optional aspect ratio locking), and cropped to eliminate excess white space. Annotations, such as subfigure labels, can be added and customized, with adjustable parameters including font type, size, weight, and color. Once assembled, the composite figure can be previewed and exported in PDF or raster formats (PNG, TIFF, JPG). Layout configurations can be saved as a template for future use. This functionality significantly reduces the technical barrier to creating multi‐panel figures, enhances efficiency, and enables users to produce figures that meet rigorous academic standards.

## FUTURE PERSPECTIVES OF THE INTEGRATED CLOUD PLATFORM FOR MICROBIOME DATA ANALYSIS

Driven by a profound understanding of global users, continuous tracking of cutting‐edge technologies, and extensive experience accrued from hundreds of thousands of projects, our team of microbiology experts has steadily enhanced and expanded our comprehensive microbial multi‐omics cloud platform suite. Our core goal remains to empower more researchers to achieve meaningful and impactful scientific outcomes. While maintaining a leading position in the field, we are committed to integrating emerging technologies into our platform—notably, mechanistic investigations of microbial epigenomics, applications of single‐cell and spatial omics in microbiology, and meta‐analysis of large‐scale microbiome datasets.

Looking ahead, AI‐driven, self‐optimizing cloud platforms will offer tremendous potential. Such systems are poised to autonomously curate high‐quality microbial databases tailored to biomedical, food, and environmental research, conduct automated data interpretation, and even propose follow‐up research strategies. In the foreseeable future, we plan to implement these advancements into the Majorbio microbial multi‐omics cloud platform.

## USERS AND PUBLICATIONS

Since October 2016, over 150,000 researchers from more than 9000 institutions worldwide have executed over 300,000 omics data mining projects on the Majorbio Cloud platform. The platform's scientific impact is evidenced by the 8000 peer‐reviewed research articles published to date that have utilized the Majorbio microbiome multi‐omics cloud platform. Notably, in 2025 alone, more than 1600 journal articles cited Majorbio Cloud. We remain committed to the continuous optimization of the platform to empower users to extract deeper biological insights from large‐scale microbiome multi‐omics data.

## AUTHOR CONTRIBUTIONS

**Jianhua Zhao**: Conceptualization; methodology; data curation; investigation; validation; formal analysis; supervision; visualization; project administration; writing—original draft; writing—review and editing. **Linmeng Liu**: Conceptualization; investigation; methodology; software; data curation; validation; formal analysis; visualization; writing—review and editing. **Jichen Han**: Conceptualization; methodology; data curation; investigation; validation; formal analysis; supervision; visualization; project administration; resources; writing—review and editing. **Junbiao Zhang**: Conceptualization; methodology; data curation; investigation; validation; formal analysis; visualization; writing—original draft; writing—review and editing. **Xiaodan Li**: Data curation; investigation; validation; formal analysis; visualization; writing—review and editing. **Yan Wang**: Data curation; investigation; validation; formal analysis; visualization; writing—review and editing. **Qianhui Han**: Data curation; investigation; validation; formal analysis; visualization; writing—review and editing. **Xue Gong**: Data curation; investigation; validation; formal analysis; visualization; writing—review and editing. **Linna Zhang**: Investigation; validation; formal analysis; data curation; visualization; writing—review and editing. **Hao Gao**: Software; data curation; investigation; validation; formal analysis; visualization; writing—review and editing. **Bing Yang**: Data curation; investigation; validation; formal analysis; writing—review and editing; visualization. **Yachen Dong**: Data curation; investigation; validation; formal analysis; visualization; writing—review and editing. **Huasheng Huang**: Resources; writing—review and editing; conceptualization. **Xianglin Zhang**: Resources; writing—review and editing; conceptualization. **Kegang Yu**: Writing—review and editing; resources; conceptualization. All authors have read the final manuscript and approved it for publication.

## CONFLICT OF INTEREST STATEMENT

The platform described in this manuscript is related to an authorized patent ZL201810796979.6. The authors are employees of Majorbio. There are no other competing interests to declare.

## ETHICS STATEMENT

No animals or humans were involved in this study.

## Supporting information


**Figure S1.** Core features of the integrated microbiome data analysis cloud platform.

**Table S1.** Systematic comparison of the integrated microbiome data analysis cloud platform on Majorbio Cloud with other platforms.
**Table S2.** Software and packages utilized in the corresponding workflows of Figure 2.
**Table S3.** Systematic comparison among three binning tools: MetaBAT2, CONCOCT, and MaxBin2.

## Data Availability

All data are available at https://cloud.majorbio.com/. Supplementary materials (figures, tables, graphical abstract, slides, videos, Chinese translated version, and update materials) may be found in the online DOI or iMeta Science http://www.imeta.science/. The data that support the findings of this study are available from the corresponding author upon reasonable request.
