# Supplementary material for: Majorbio Cloud 2026 provides comprehensive analysis workflows for microbiome
Source: Imeta. 2026 Feb 5;5(1):e70109. doi: 10.1002/imt2.70109 (PMC13726581; doi:10.1002/imt2.70109)
Supplement: Supplementary file 1 [file IMT2-5-e70109-s002.docx]

**Supporting information to**

**Majorbio Cloud provides comprehensive analysis workflows for microbiome**

**Running title:** Microbiome analysis workflows on Majorbio Cloud

Jianhua Zhao^#*^, Linmeng Liu^#^, Jichen Han^#*^, Junbiao Zhang^#^, Xiaodan Li, Yan Wang, Qianhui Han, Xue Gong, Linna Zhang, Hao Gao, Bing Yang, Yachen Dong, Huasheng Huang, Xianglin Zhang, Kegang Yu

Shanghai Majorbio Bio-pharm Technology Co., Ltd, Shanghai 200120, China

^#^These authors contributed equally: Jianhua Zhao, Linmeng Liu, Jichen Han, and Junbiao Zhang

^*^Correspondence: [jichen.han@majorbio.com](mailto:jichen.han@majorbio.com) (Jichen Han), [jianhua.zhao@majorbio.com](mailto:jianhua.zhao@majorbio.com) (Jianhua Zhao)

**Supplementary methods**

**Detailed descriptions of the eight core workflows and two cross-omics integration workflows**

This integrated platform features eight core workflows spanning key domains of microbiome research, enabling precise and in-depth analysis of each data type: (1) Bacterial (Archaeal)/Fungal genome**:** Tailored for pure cultures, this workflow facilitates genome assembly, functional annotation, and comparative genomics; (2) Prokaryotic transcriptomics: Profiles genome-wide transcriptional dynamics in prokaryotes, elucidating active biological processes under defined experimental conditions; (3) Amplicon sequencing: Leveraging marker genes (e.g., 16S/18S rRNA or ITS), this workflow enables high-resolution profiling of microbial community composition and taxonomic diversity; (4) Metagenomics: Employs shotgun sequencing for the comprehensive characterization of taxonomic composition, gene catalogs, and functional potential of microbial communities; (5) Metagenome-assembled genom**e**: Facilitates the recovery of microbial genomes from metagenomic datasets, enabling the identification of uncultured microorganisms and their metabolic potential; (6) Metatranscriptomics: Directly quantifies community-level transcriptional activity, identifying functionally active genes at specific time points or under defined experimental treatments; (7) Proteomics: Identifies and quantifies the complete proteome expressed by a microbial community, revealing the functional executors of microbial metabolism; (8) Metabolomics: Identifies and quantifies small-molecule metabolites produced or regulated by the microbiome, providing a direct snapshot of community-level metabolic activity.

To overcome the limitations of single-omics strategies, this platform has incorporated advanced cross-omics integration workflows. These workflows are specifically designed to elucidate intrinsic correlations across distinct molecular layers. (1) Microbiome-metabolome association analysis: This workflow integrates microbial community composition data (e.g., from amplicon sequencing) and/or functional gene profiles (e.g., from metagenomic) with metabolite abundance data. By bridging the gap between microbial taxonomy (“who is present”) and metabolic output (“what they are doing”), it unravels the mechanistic pathways through which microbiome modulate host or environmental phenotypes. (2) Microbiome-host transcriptome association analysis: This workflow facilitates the systematic integration of host transcriptome data (e.g., from humans, animals, or plants) with microbiome profiles to decipher the functional crosstalk between microbial communities and host physiology. It is tailored to uncover the mechanistic underpinnings of processes such as immunity, metabolism, and development, elucidating the bidirectional regulatory networks that govern host-microbe interactions.

In summary, this integrated platform offers a unified analytical framework for microbiome research, bridging the gap between descriptive community ecology and causal, mechanism-driven insights—a pivotal step toward advancing functional microbiome science.

**Other representative cloud-based workflows for microbiome data analysis**

Example 1: Bacterial genomic analysis workflow

As a cornerstone of microbiology, bacterial genomes encode the complete genetic information of bacteria, laying the foundation for elucidating biological characteristics, pathogenic mechanisms, evolutionary relationships, and industrial applications. Bacterial genome studies are primarily categorized into two types—draft genomes and complete genomes—depending on the required completeness and accuracy thresholds. To address these distinct requirements, we have developed three complementary workflows (Figure 1): the Draft genome workflow, the Complete genome workflow, and the Comparative genomic workflow.

The Draft Genome Workflow facilitates high-throughput, cost-effective sequencing and functional screening of large bacterial strain collections. In contrast, the Complete Genome Workflow employs long-read sequencing technologies to generate gap-free, complete genomes, yielding high-quality reference sequences for detailed investigations of genomic architecture. Building on these foundations, the Comparative Genomic Workflow enables systematic cross-genome analyses to infer evolutionary trajectories and genetic determinants underlying phenotypic diversity.

For genome assembly, this cloud-based workflow supports multiple strategies. Unicycler [1] is available for hybrid assembly of short-read (second-generation) and long-read (third-generation) sequencing data. Alternatively, specialized tools such as Canu [2], Flye [3], and NextDenovo [4] are dedicated to assembling long-read (third-generation) sequencing data alone, followed by polishing with Pilon [5] and short-read sequencing data to improve assembly accuracy.

For Gene prediction, the workflow integrates specialized tools: Prodigal [6] for prokaryotic gene prediction, GeneMarkS [7] for plasmid-borne gene prediction, tRNAscan-SE 2.0 [8] for tRNA gene prediction, and Barrnap v0.9 for rRNA gene detection.

For functional annotation, predicted genes are aligned against a comprehensive panel of 17 widely used databases. These include general-purpose resources (e.g., NR, Pfam, eggNOG, GO, KEGG and CAZy) , as well as specialized databases focusing on pathogenic traits (e.g., VFDB, CARD, PHI-base, TCDB, and QSDB).

Finally, the workflow generates comprehensive genomic visualizations (e.g., using CGview and Circos) as part of its standard output.

Example 2: Metatranscriptomic analysis workflow

In the investigation of environmental microorganisms, merely characterizing “who is present” (metagenomics) is insufficient; therefore, understanding “what they are actively engaged in” (metatranscriptomics) is paramount. As a pivotal technology for probing gene expression in microbial communities, metatranscriptomics circumvents the need for microbial cultivation by directly analyzing community mRNA, enabling direct characterization of active community members, their expressed genes, and corresponding functions [9]. Thus, metatranscriptomics uncovers biological insights that are not readily accessible via conventional genomic profiling approaches, complementing marker gene amplicon sequencing and metagenomic analyses—approaches that typically cannot distinguish among active, inactive, and non-viable community members.

In July 2024, we launched the metatranscriptomic analysis workflow, which offers both reference-free and metagenome-guided approaches, the latter leveraging prior metagenomic assemblies for improved annotation (Figure 2). The subsequent sections will focus on the reference-free metatranscriptomic analysis approach.

The workflow comprises seven sequential core steps: data preprocessing, transcriptome assembly, gene prediction, construction of non-redundant gene sets, gene expression quantification, differentially expressed gene (DEG) analysis, taxonomic and functional annotation, and data visualization. It integrates nine annotation databases, namely NR, eggNOG, GO, KEGG, CAZy, CARD, BacMet, VFDB, and PHI-base. Additionally, the workflow supports analysis using eleven specialized functional gene sets (e.g., those associated with key metabolic pathways). For human microbiota research, it incorporates advanced analytical modules including Random Forest, Receiver Operating Characteristic (ROC) analysis, and Weighted Gene Co-expression Network Analysis (WGCNA).

To date, this workflow has supported users in publishing 123 scientific articles (based on a Google Scholar search conducted on December 3, 2025, using the search term: “cloud.majorbio.com metatranscriptomics”). Future developments will focus on optimization and the establishment of MAG-centric metatranscriptomic analysis workflows.

**References**

1. Wick, Ryan R, Louise M Judd, Claire L Gorrie, Kathryn E Holt. 2017. “Unicycler: resolving bacterial genome assemblies from short and long sequencing reads.” *PLoS Computational Biology* 13: e1005595. <https://doi.org/10.1371/journal.pcbi.1005595>

2. Koren, Sergey, Brian P Walenz, Konstantin Berlin, Jason R Miller, Nicholas H Bergman, Adam M Phillippy. 2017. “Canu: scalable and accurate long-read assembly via adaptive k-mer weighting and repeat separation.” *Genome Research* 27: 722−736. <https://doi.org/10.1101/gr.215087.116>

3. Kolmogorov, Mikhail, Jeffrey Yuan, Yu Lin, Pavel A. Pevzner. 2019. “Assembly of long, error-prone reads using repeat graphs.” *Nature Biotechnology* 37: 540−546. <https://doi.org/10.1038/s41587-019-0072-8>

4. Hu, Jiang, Zhuo Wang, Zongyi Sun, Benxia Hu, Adeola Oluwakemi Ayoola, Fan Liang, Jingjing Li, José R Sandoval, David N Cooper, Kai Ye. 2024. “NextDenovo: an efficient error correction and accurate assembly tool for noisy long reads.” *Genome Biology* 25: 107. <https://doi.org/10.1186/s13059-024-03252-4>

5. Walker, Bruce J, Thomas Abeel, Terrance Shea, Margaret Priest, Amr Abouelliel, Sharadha Sakthikumar, Christina A Cuomo, Qiandong Zeng, Jennifer Wortman, Sarah K Young. 2014. “Pilon: an integrated tool for comprehensive microbial variant detection and genome assembly improvement.” *PloS One* 9: e112963. <https://doi.org/10.1371/journal.pone.0112963>

6. Hyatt, Doug, Gwo-Liang Chen, Philip F. LoCascio, Miriam L. Land, Frank W. Larimer, Loren J. Hauser. 2010. “Prodigal: prokaryotic gene recognition and translation initiation site identification.” *BMC Bioinformatics* 11: 119. <https://doi.org/10.1186/1471-2105-11-119>

7. Besemer, John, Alexandre Lomsadze, Mark Borodovsky. 2001. “GeneMarkS: a self-training method for prediction of gene starts in microbial genomes. Implications for finding sequence motifs in regulatory regions.” *Nucleic Acids Research* 29: 2607−2618. <https://doi.org/10.1093/nar/29.12.2607>

8. Chan, Patricia P, Brian Y Lin, Allysia J Mak, Todd M Lowe. 2021. “tRNAscan-SE 2.0: improved detection and functional classification of transfer RNA genes.” *Nucleic Acids Research* 49: 9077−9096. <https://doi.org/10.1093/nar/gkab688>

9. Ojala, Teija, Aino-Elina Häkkinen, Esko Kankuri, Matti Kankainen. 2023. “Current concepts, advances, and challenges in deciphering the human microbiota with metatranscriptomics.” *Trends in Genetics* 39: 686−702. <https://doi.org/10.1016/j.tig.2023.05.004>

**Supplementary figures**

**Figure S1 Core features of the integrated microbiome data analysis cloud platform**. This figure outlines four core features: (1) Application guide; (2) Default analysis parameters and one-click Analysis; (3) One-click plot enhancement; (4) Plot patchwork feature. Users can customize analytical workflows, perform one-click analysis via pre-optimized default parameters, select corresponding plot templates to refine visualization results, store publication-ready images in My gallery, and leverage the Plot patchwork feature for flexible image assembly—seamlessly achieving integration from data analysis to result presentation.
